# One-Step Pyrolysis of Nitrogen-Containing Chemicals and Biochar Derived from Walnut Shells to Absorb Polycyclic Aromatic Hydrocarbons (PAHs)

**DOI:** 10.3390/ijms232315193

**Published:** 2022-12-02

**Authors:** Wendong Wang, Donghua Li, Ping Xiang, Yunwu Zheng, Zhifeng Zheng, Xu Lin, Xiahong He, Can Liu

**Affiliations:** 1 National Joint Engineering Research Center for Highly-Efficient Utilization Technology of Forestry Resources, Southwest Forestry University, Kunming 650224, China; 2 College of Chemical Engineering, Huaqiao University, Xiamen 361021, China

**Keywords:** walnut shell, pyrolysis, nitrogenous chemicals, PAHs, biochar

## Abstract

The pyrolysis of biomass is an efficient means of utilizing biomass resources. Biomass can be converted into various high-performance chemicals and functional materials through pyrolysis. However, current pyrolysis technologies suffer from low conversion rates and single products, so the preparation of nitrogen compounds with high economic value remains a challenge. The walnut shell was soaked in three nitrogen-containing compound solutions before carbonization to produce high-value-added nitrogen-containing chemicals (with a nitrogen content of 59.09%) and biochar for the adsorption of polycyclic aromatic hydrocarbons (PAHs). According to biochar analysis, biochar has a porous structure with a specific surface area of 1161.30 m^2^/g and a high level of rocky desertification. The surface forms a dense pyrrole structure, and the structure produces π–π interactions with naphthalene molecules, exhibiting excellent naphthalene adsorption with a maximum capacity of 214.98 mg/g. This study provides an efficient, rapid, and environmentally friendly method for producing nitrogen-containing chemicals with high-added value and biochar.

## 1. Introduction

The pharmaceutical, pesticide, and dye industries extensively use nitrogen compounds, particularly pyrroles, pyridines, and indoles [1]. Currently, nitrogen compounds are primarily produced by oxidizing petroleum hydrocarbons with ammonium. Typically, the treatment process is associated with numerous pollutants. Numerous issues exist, including high production costs, low efficiency, poor selectivity, complex catalytic systems, and lengthy and intricate reaction steps. From this perspective, an environmentally friendly, efficient, and practical preparation method for synthesizing nitrogenous compounds from inexpensive and readily available starting materials is crucial [2,3]. In addition, some researchers have discovered that biomass containing more oxygen groups is more likely to be ammoniated, producing more nitrogen-containing chemicals than hydrocarbons derived from petroleum [4]. Therefore, catalytic pyrolysis of biomass is an essential method for producing high-value-added chemicals. Utilizing pyrolytic biomass to produce chemicals with high-added value is therefore renewable, abundant, carbon neutral, and environmentally friendly. By judiciously adjusting the reaction process, biomass can be utilized to produce nitrogenous chemicals with vast application potential.

The class of organic pollutants known as polycyclic aromatic hydrocarbons (PAHs) is prevalent in water and soil. Compounds resulting from condensing two or more benzene rings [5], PAHs have powerful carcinogenic, teratogenic, and mutagenic effects on living organisms. The Environmental Protection Agency (EPA) of the United States has 16 types of PAHs listed as priority pollutants and under environmental control [6]. They can be divided into light polycyclic aromatic hydrocarbons (≤4 benzene rings) and heavy PAHs (>4 benzene rings) based on the number of benzene rings. When the number of benzene rings in PAHs is high, their hydrophobicity, stability, and toxicity are also high [7], which prevents PAHs from being directly removed by conventional methods, such as filtration, flocculation, ozone oxidation, coagulation, and precipitation [8,9]. Adsorption materials, such as biochar [10,11,12], activated clay [13], and adsorption resin [14], have been widely used to remove PAHs, whose pollution is becoming increasingly severe. Biochar has attracted considerable interest due to its vast surface area, abundant pore structure, and low cost [15]. Due to its lower polarity and higher aromaticity, Zhu et al. [16] prepared oleic acid-grafted walnut shells to absorb naphthalene in solution; the naphthalene adsorption amount was 7.21 mg/g. Researchers found that after modifying coal-activated carbon with microwave-irradiated iron, the amount of oxygen-containing groups on the surface of activated carbon decreased, the alkalinity increased, and the PAH adsorption capacity increased significantly. The adsorption capacity of naphthalene was 160.88 mg/g [17]. Cellulose, hemicellulose, and lignin make up most walnut shells. The surface contains many oxygen-containing functional groups and is an excellent carbon source [18,19]. The adsorption of PAHs by activated carbon is currently plagued by numerous issues, including high preparation costs, low adsorption efficiencies, and an unknown adsorption mechanism. To address the issues above, developing a green, efficient, and practical method for preparing activated carbon from walnut shells is necessary.

**Figure 1:**
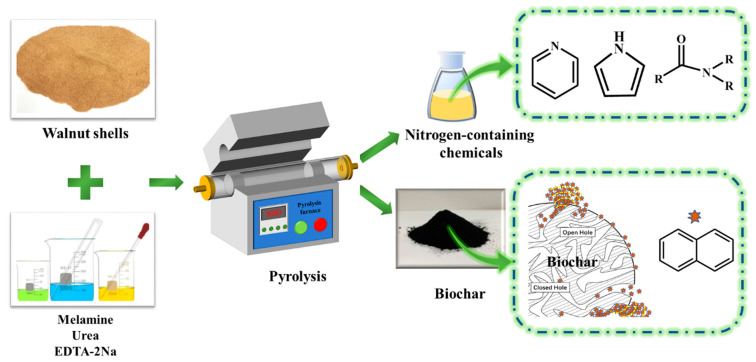
Schematic diagram of one-step preparation of nitrogenous chemicals and biochar.

Herein, we describe a type of walnut shell as a raw material and the impregnation of walnut shell powder with melamine, urea, and EDTA-2Na as nitrogen sources in a specific ratio. The impregnated biomass was then pyrolyzed in a fixed-bed reactor under a N_2_ atmosphere, and the gases from the pyrolysis process were found to contain high levels of nitrogenous chemicals. Surprisingly, when residual biochar was analyzed for PAH (naphthalene) adsorption experiments, it was discovered that the biochar had a greater adsorption effect on naphthalene (Figure 1). This method of preparing nitrogen-containing chemicals and biochar (for PAH removal) in a single step by impregnation with a nitrogen source and copyrolysis provides efficient and convenient access to nitrogen-containing chemicals and high-value biochar. This may be a novel way to utilize valuable walnut shells.

## 2. Results

### 2.1. Pyrolysis Product Analysis

**Figure 2:**
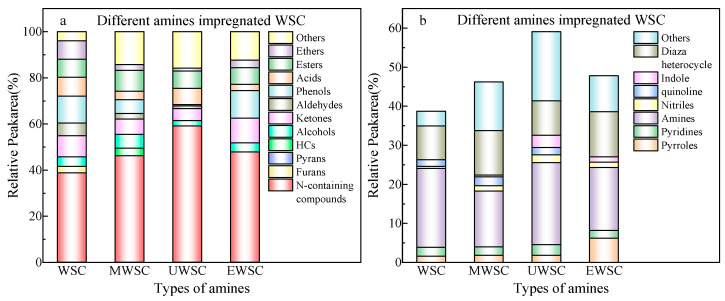
Product yield and N-containing compounds distribution of pyrolysis bio-oil derived WSC. (**a**) Yield of nitrogen-containing compounds; (**b**) Selectivity of nitrogen-containing compounds.

The composition of the bio-oil products was analyzed to determine the effect of ammonia source immersion on the bio-oil products; the results are shown in Figure 2. The yield and selectivity of nitrogenous compounds in chemicals are shown in Figure 2 because of soaking walnut shells in various ammonia sources. The organic phases of walnut shell pyrolysis products can be categorized into 12 groups, which include nitrogen compounds, furans, pyrans, hydrocarbons, alcohols, ketones, aldehydes, phenols, acids, esters, and ethers. After soaking, the content of nitrogen compounds in a solution of disodium ethylenediaminetetraacetate, melamine, and urea increased significantly, with urea constituting the highest nitrogen source at 59.09%. An alkaline urea solution at high temperatures accelerates the dissociation of cellulose and hemicellulose, thereby facilitating the entry of N elements into the interior. It may react with aldehydes, ketones, acids, furans, and other carbonyl compounds produced by the pyrolysis of walnut shells, thereby increasing nitrogen-containing compounds. The oxygenates and NH_3_ produced by the thermal decomposition of urea can generate nitrogenous compounds via dehydration, dehydrogenation, decarbonylation, condensation, cyclization, and the Maillard reaction [20]. The selectivity of nitrogen-containing compounds is depicted in Figure 2b. Compounds containing nitrogen consist primarily of pyrrole, pyridine, amines, and diazo heterocycles. The most abundant compounds are amines, predominantly amides formed by the Maillard reaction [21,22].

### 2.2. Pore Structure Analysis

**Figure 3:**
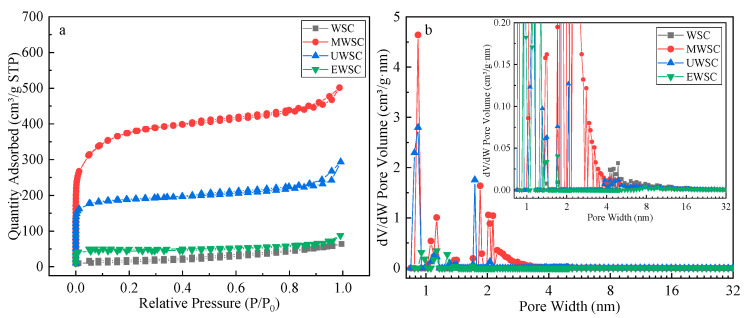
N_2_ adsorption-desorption isotherms (**a**) and pore size distribution (**b**) of activated carbon.

As depicted in Figure 3, the N_2_ adsorption-desorption isotherm of the four biochar types is a type IV adsorption isotherm with an H_4_-type hysteresis ring, demonstrating the micromesoporous adsorption characteristics [16]. The Type IV adsorption isotherm rises rapidly at lower relative pressures, and the curve is convex; at higher P/P_0_, the adsorption increases slightly with relative pressure. As the saturation pressure reaches the medium, a specific hysteresis back loop appears, which corresponds to the system of capillary coalescence of the porous adsorbent. After the mesoporous capillary coalescence fills up, the adsorption isotherm continues to rise. This indicates that the biochar likewise has large diameter pores or a strong adsorbent molecule interaction, potentially forming multimolecular layer adsorption, a typical capillary coalescence phenomenon, multilayer adsorption, and eventually a type IV adsorption isotherm. The hysteresis loop of H_4_ indicates that this biochar’s pore size comprises a mixture of microporous and mesoporous pores with narrow fissure pores [23].

As shown in Table 1, adding nitrogen sources increased the S_BET_, V_total_, and V_mic_ of biochar. The coactivation of a nitrogen source will corrode the carbon layer, causing the pores within the biochar to enlarge, thereby increasing the S_BET_ and V_total_ capacity and decreasing the average pore size (D_ap_). The S_BET_, V_total_, and V_mic_ values of the four biochars were ranked MWSC > UWSC > EWSC > WSC, whereas the average pore size was ranked in the exact opposite order. SBET (1161.30 m^2^/g) and V_total_ (0.7128 cm^3^/g) were the greatest for biochar prepared with melamine as the nitrogen source. The high mesoporous ratio of MWSC facilitates the formation of capillary condensation, enabling multilayer adsorption of an adsorbent. D_ap_ defines the ability of adsorbed molecules to permeate within the biochar [24]. For adsorbed molecules to permeate the adsorbent, the pore diameter must have a larger effective molecular diameter than the adsorbate. The effective molecular diameter of naphthalene is approximately 1.16 nm; so theoretically, all four biochars are suitable for naphthalene adsorption.

### 2.3. SEM Analysis of Apparent Morphology

**Figure 4:**
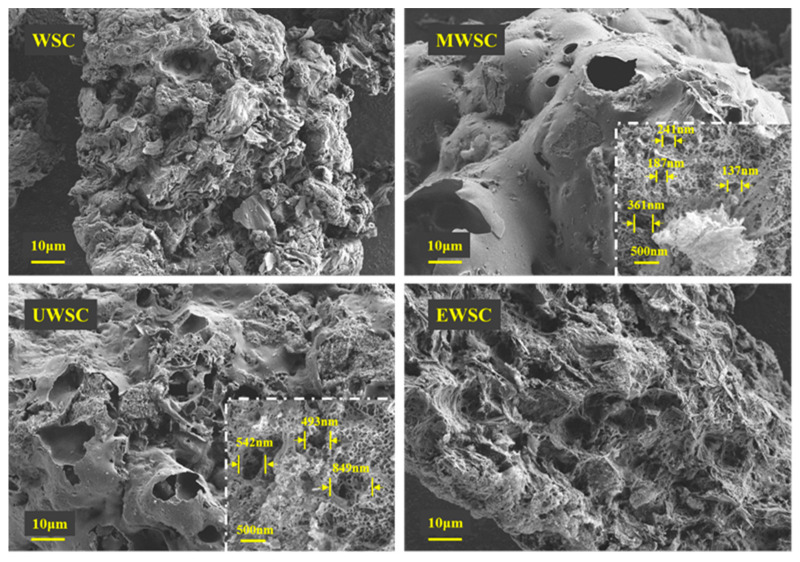
SEM of the surface structure of activated carbon.

Figure 4 shows that impregnation and pyrolysis can alter the surface structure of biochar. If biochar is not modified, its surface is rough, there are more minute particles, and the pore structure is diminished. Nitrogen doping decreased the small surface particles of biochar and increased the surface pore density of the modified biochar. As shown in Table 1, the increase in porosity density results in a rise in specific surface area, resulting from the nitrogen source’s further corrosion of the carbon layer. In contrast, the surface of MWSC was composed of an abundance of macropores ranging in size from 361 to 137 nm. The corrosion of the internal pore structure, resulting in a partial collapse of the pore channels and, thus, an increase in pore size, led to numerous voids on the surface of UWSC, which was comparable to that of biochar. According to BET data, the pore size of UWSC was greater than that of MWSC, and a significant number of pores on the surface of EWSC collapsed, resulting in surface depression. According to the BET data, its specific surface area was only 136.89 m^2^/g, while its Dap was 3.19 nm, the largest pore size of the three biochars.

### 2.4. XRD Analysis of Biochar

**Figure 5:**
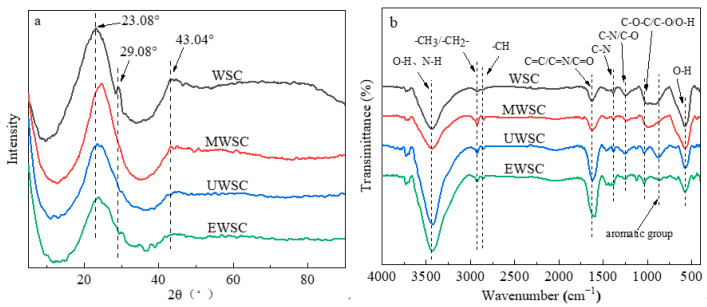
XRD (**a**) and FTIR (**b**) spectra of activated carbon samples.

Four biochars were subjected to X-ray diffraction analysis, and the crystalline structures are depicted in Figure 5a. Accordingly, all samples exhibit diffraction peaks at approximately 2θ values of 23° and 43°. The 23° crystalline plane of carbon material 002 is one of them. The 43° is crystallographic plane 100 of the graphite structure, which exhibits microcrystalline characteristics. Furthermore, near 29° is the peak of the oxide of elemental Si in walnut shells, where the 29° peak appears only on WSC and disappears after the activation of nitrogen chemical agents. These agents facilitate the deashing of biochar, resulting in the disappearance of the peak of elemental Si near 29°. The analysis of diffractograms reveals that the graphitized carbon peak at 43° is weaker than the peak at 23°, indicating that the graphitization of biochar is generally low and amorphous carbon predominates in biochar. The addition of nitrogen had no significant effect on the carbon material’s skeletal structure.

As shown in Figure 5b, the carbon material was characterized by Fourier transform infrared spectroscopy (FT-IR) [25,26,27,28]. As shown in the figure, both the C-O-C and C-O peaks were attenuated following nitrogen doping of the biochar, indicating that nitrogen doping played a more significant role in deoxygenation. Concurrently, both UWSC and EWSC exhibit a relatively distinct (C=C) aromatic ring characteristic peak at 870 cm^−1^, indicating the formation of a portion of graphitized structured carbon.

### 2.5. XPS Analysis of Biochar

**Figure 6:**
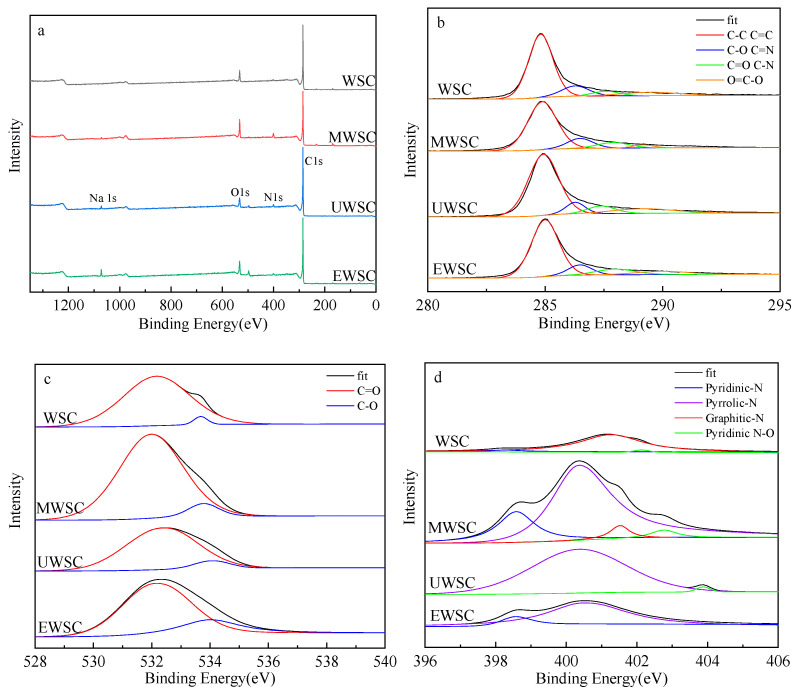
XPS patterns of Total (**a**), C 1s (**b**), O1s (**c**), N 1s (**d**).

As shown in Figure 6a, XPS scanning spectroscopy detected three prominent peaks of C 1s, O 1s, and N 1s on the biochar surface, with C 1s (285 eV) and O 1s (532 eV) predominating in the biochar sample. Table 2 lists the relative atomic contents, with the highest nitrogen atom content at 6.25% (MWSC). The carbon content of biochar decreased when nitrogen was added, while the N content increased. The relative contents of elemental N on the surface of the biochar were in the order of MWSC > UWSC > EWSC > WSC. Meanwhile, the oxygen contents of the biochar were ranked as MWSC > EWSC > WSC > UWSC. Oxygen-containing groups can increase the hydrophilicity of carbon materials [28], so the hydrophilicity of carbon materials is consistent with the oxygen content ranking. The UWSC biochar has the lowest oxygen content and the highest hydrophobicity, indicating that the superior deoxygenation effect of urea activation can significantly reduce the content of polar groups on the surface of biochar, which can interact with hydrophobic naphthalene molecules and promote adsorption. Additionally, it is known that MWSC carbon has a lower C content and a relatively higher N and O content.

Different binding energies in the C1s spectrum correspond to various forms of C elements. Peaks near 284.8, 286.5, 287–288, and 289.5 eV correspond to C-C/C=C, C-O/C=N, C=O/O-C-O/C-N, and O=C-O, respectively [29,30]. Each structure’s proportion is listed in Table 3 for the C element on the surface of the four biochars. According to the results, the relative content of C-C/C=C in MWSC materials was the highest at 73.98%. This result indicates that the biochar surface formed more graphitic and aromatic structures that could generate π–π interactions with the benzene ring structure of naphthalene molecules, which was favorable for adsorption. Moreover, its O=C-O content was the lowest, indicating that melamine has a superior deoxidation effect compared to the other two modifiers. The modified biochar’s C=O/C-N relative contents were significantly increased, indicating that nitrogen source doping could load the biochar surface with N elements. Such special electron cloud characteristics of the biochar surface could quickly generate p–π interactions with aromatic ring-like substances. These could provide lone pairs of electrons and π electrons for the reaction, thus facilitating naphthalene adsorption.

Figure 6c and Table 4 illustrate the relative proportion of the oxygen element bonding types. In all samples, the O=C content was greater than the O-C content. During activation, the lignin in the walnut shell underwent oxidation and condensation reactions, resulting in many carbonyl groups, thereby increasing the O=C peak area of biochar [31]. Comparatively, the MWSC material contains more oxygen bonds that are not polar.

The N elements on the biochar surface were analyzed. Table 5 reveals three structural types of N on the surface of WSC biochar, namely, pyrrole-N, graphite-N, and oxide-N, with the graphite-N structure predominating, among which MWSC and UWSC pyrrole-N contents are relatively high. The unique structure of pyrrole-N produces π–π interactions with naphthalene molecules, which facilitates adsorption [32].

### 2.6. Effects of Adsorption Process Conditions on the Adsorption Properties of Naphthalene

**Figure 7:**
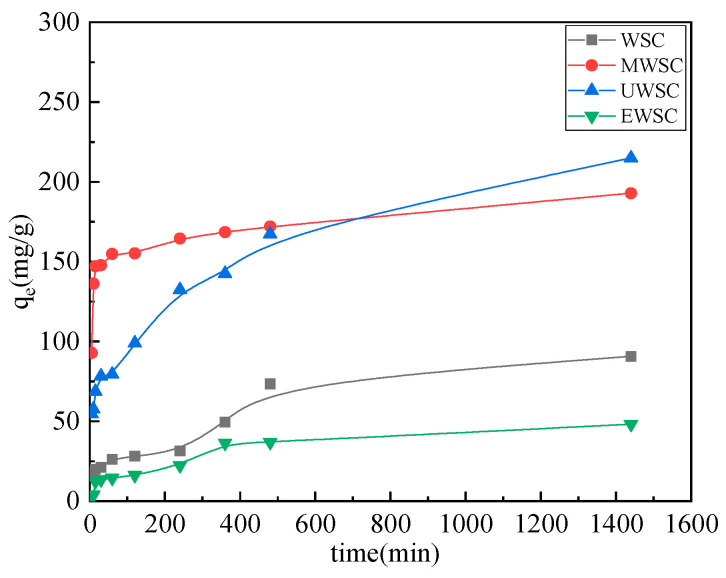
Effect of adsorption time on the Naphthalene adsorption capacity of activated carbon.

As shown in Figure 7 and Table 6, the biochar’s naphthalene adsorption capacity increased as the adsorption time increased. During the initial phase of the naphthalene adsorption reaction, biochar had a high adsorption capacity and a rapid adsorption rate. Although the adsorption capacity increased slightly as the adsorption time increased continuously, the change was subtle and tended to be flat. This is primarily attributable to the abundance of functional groups on the surface of biochar, the high concentration of naphthalene in the solution during the earliest stage of pore development, and the high mass transfer driving ability, which results in the highest adsorption rate. At 480 min, the adsorption rate decreased during the adsorption process as the concentration of naphthalene decreased and the driving force was reduced; at more than 480 min, the adsorption amount of adsorption essentially did not change and tended toward adsorption-desorption dynamic equilibrium. After coactivation with a nitrogen source, the naphthalene adsorption capacity of biochar (MWSC/UWSC) increased significantly. The biochar saturable adsorption capacities were UWSC > MWSC > WSC > EWSC, with UWSC reaching a maximum value of 214.98 mg/g. The S_BET_, V_total,_ and V_mic_ values of UWSC were larger, while those of D_ap_ were also larger than those of MWSC, making it easier for naphthalene molecules to enter the biochar and conducive to adsorption. Moreover, the surface was abundant in nitrogen-containing functional groups, such as C-N, C=N, pyrrole-N, and pyridine-N. These groups enhance the chemical groups on the surface and facilitate the bonding and interaction between the biochar and the benzene ring structure of naphthalene molecules for adsorption [32], resulting in the greatest naphthalene adsorption capacity. The surface functional groups of WSC and EWSC are comparable, but the mesopore pore capacity of WSC is greater than that of EWSC, resulting in greater naphthalene adsorption by WSC.

According to Table 7, the fitting correlations of the biochar pseudo-second-order kinetic equations are all greater than those of the biochar pseudo-first-order kinetic equations. In the pseudo-second-order kinetic equations, the correlation between the adsorption of naphthalene by biochar was R^2^ > 0.893. As determined by pseudo-second-order kinetics, naphthalene’s theoretical adsorption value q_e_ is close to the experimental adsorption amount q_exp_. Therefore, the solution adsorption of naphthalene molecules by biochar is consistent with the pseudo-second-order kinetic equation. The results indicate that biochar can adsorb naphthalene in two ways, physical and chemical, with chemisorption predominating.

**Figure 8:**
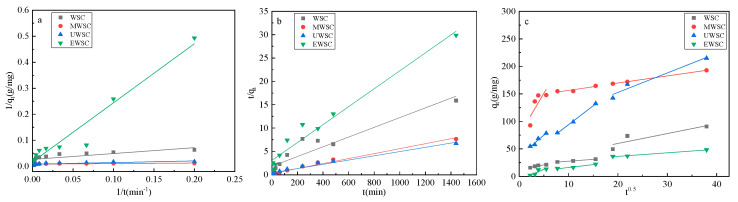
Fitting curve of activated carbon adsorption kinetics: Pseudo-first-order kinetic (**a**), Pseudo-second-order kinetic (**b**), and Webber and Morris model (**c**).

When the fitted linear curve does not pass through the origin, the results of fitting the intraparticle diffusion equation indicate that two or more diffusion mechanisms are the primary factors controlling the adsorption rate. As shown in Figure 8c and Table 8, there is no linear correlation between q_t_ and t^0.5^; instead, three linearly correlated lines were fitted, indicating that some degree of boundary layer control was involved in the naphthalene adsorption history by biochar [33]. The process of overcoming the adsorption energy barrier of naphthalene molecules from the solution to the biochar surface is depicted in the first line. The second straight line depicts the path of naphthalene molecules penetrating and diffusing from the surface of biochar into the interior of the pores. The third straight line depicts the biochar’s naphthalene adsorption, tending toward an equilibrium between adsorption and desorption. As demonstrated by the above results, the adsorption rate is governed by numerous factors, including particle diffusion, surface adsorption, and liquid film diffusion.

**Figure 9:**
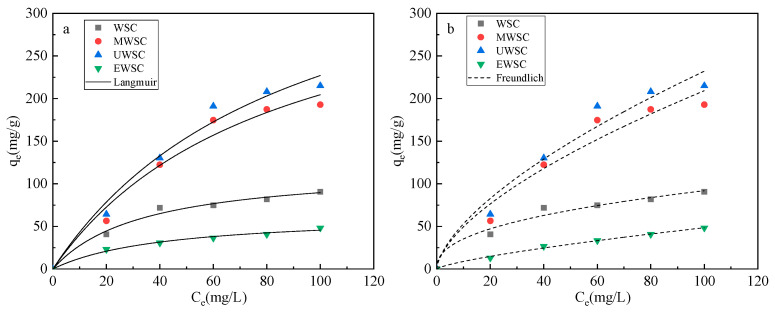
Isothermal fitting adsorption curve of activated carbon: Langmuir (**a**) and Freundlich (**b**) isothermal fitting curve.

Figure 9 and Table 9 illustrate the adsorption isotherm model parameters fitted analytically to the naphthalene adsorption data of biochar by utilizing the Langmuir and Freundlich equations. From the results, the correlation between biochar and the Freundlich equation is higher than that between biochar and the Langmuir equation. This indicates that the adsorption of naphthalene by N-doped biochar was more consistent with the Freundlich model and was dominated by multimolecular layer adsorption, which refers to the phenomenon of the readsorption of adsorbed mass molecules. The larger the value of K_F_ is, the stronger the adsorption of naphthalene, and the order of the adsorption strength of naphthalene by biochar is as follows: WSC > UWSC > MWSC > EWSC. When the value of n is high, naphthalene is more readily adsorbed by biochar. When n is less than 1, it indicates an unfavorable adsorption process; when 1 < n < 2, it indicates a favorable adsorption process; when 2 < n < 10, it likewise denotes a favorable adsorption process. According to Table 9, the adsorption of naphthalene by WSC and EWSC biochar was favorable, whereas the adsorption of naphthalene by MWSC and UWSC biochar was preferable. From the Langmuir equation fitting results, the order of the saturated theoretical adsorption was UWSC > MWSC > WSC > EWSC, while the theoretical maximum adsorption was 431.02 mg/g, consistent with the order of magnitude of the experimental adsorption of naphthalene.

## 3. Discussion

We copyrolyzed biomass with three nitrogen sources (urea, EDTA-2Na, and melamine) to produce nitrogenous chemicals and biochar. Most chemical compounds contain pyrrole, pyridine, amine, and diazo heterocyclics. When urea was used as the nitrogen source, the relative concentration of nitrogenous chemicals was the highest (59.09%). The analysis of residual biochar following pyrolysis revealed that the specific surface area of the biochar formed after nitrogen doping increased and that the specific surface area of MWSC could reach 1161.30 m^2^/g. The addition of urea and melamine resulted in biochar with a relatively high graphitization structure, as well as a rich pyrrole structure and carbonyl bond on the surface. Both MWSC and UWSC naphthalene exhibited effective adsorption properties, and these groups facilitated the binding and interaction between the biochar and the benzene ring structure of the naphthalene molecule. UWSC reached 214.98 mg/g, while MWSC reached 192.85 mg/g. The adsorption of naphthalene by biochar closely resembles the second-order kinetic equation, which describes a mixture of chemisorption and physisorption. The parameters of the adsorption isotherm model indicate that multimolecular layer adsorption predominates in naphthalene adsorption. The one-pot method produces high-value nitrogen-containing chemicals and biochar with high adhesion, providing a new strategy for efficiently utilizing biomass.

## 4. Materials and Methods

### 4.1. Materials

Walnut shell is taken from Yangbi County, Dali City, Yunnan Province. We dried it at 80 °C for 24 h, crush and pass through a 100-mesh sieve and set it aside in an airtight container. Naphthalene, NaHCO_3_, melamine, urea, EDTA-2Na (AR) and ethanol (99.7%) were provided by Shanghai Tai’an Technology Co., Ltd. (Shanghai, China). Unless otherwise stated, all reagents are used as is and no further purification is required. Deionized (DI) water was used throughout the study.

### 4.2. Methods

Analysis of chemicals: The column used in gas chromatography mass spectrometry (GC-MS) was an HP-5MS capillary column of itq900 (Thermo Fisher Scientific, Waltham, MA, USA). The chemical composition of the bio-oil was analyzed by a semi-quantitative method. One hundred fractions were selected, and the content of each fraction was calculated by the area normalization method. Characterization of Biochar: Surface specifications were to use ASAP2020 surface specifications and pore size analyzer (Micromeritics, Norcros, GA, USA) measurement. BET equation, BJH method, and density function theory were used to calculate the specific surface area (S_BET_) and micropore volume (V_mic_) of biochar. Zeiss Gemini 300 scanning electron microscope (SEM) was used to analyze the surface morphology of biochar. The degree of crystallization of the biochar was tested using an X-ray diffractometer at Brock D8 ADVANCE. FTIR analysis was performed using a MagnaIR-560E.S.P infrared spectrometer (Nicolet, Madison, WI, USA). X-ray photoelectron spectroscopy (XPS) was used to determine the surface of the biochar C, N, O, and valence. Shimadzu UV-vis spectrum was used to determine the absorbance of the naphthalene solution.

### 4.3. Preparation of Biochar and Nitrogen-Containing Chemicals

In the synthesis process, the previously prepared walnut shell powder was mixed with nitrogen sources (melamine, urea, EDTA-2Na) and macerated at 80 °C under magnetic stirring until dry. The former walnut shell powder was added to the fixed-bed pyrolysis reactor, heated at 5 °C/min under an N2 atmosphere, and pyrolyzed at 600 °C for 30 min. We used water and ethanol to rinse the biochar to neutral and then dried it at 80 °C. Biochar prepared by pyrolysis of simple walnut shell powder was named WSC, the melamine-impregnated biochar was named MWSC, the urea-impregnated biochar was named UWSC, and the EDTA-2Na impregnated biochar was named EWSC.

Nitrogen-containing chemicals acquisition method: Our experimental setup was mainly composed of three parts, namely, pyrolysis reactor, catalytic reactor, and condensing tube [34]. The reactor was electrically heated, and the temperature was internally measured by thermocouples. Pyrolysis and catalytic pyrolysis reactions were carried out in a reactor. A catalytic pyrolysis experiment was conducted under the environment of nitrogen and kept for 30 min. Finally, nitrogenous chemicals were collected at the tail of the unit and analyzed using GC/MS.

### 4.4. Standard Curve Plotting

**Figure 10:**
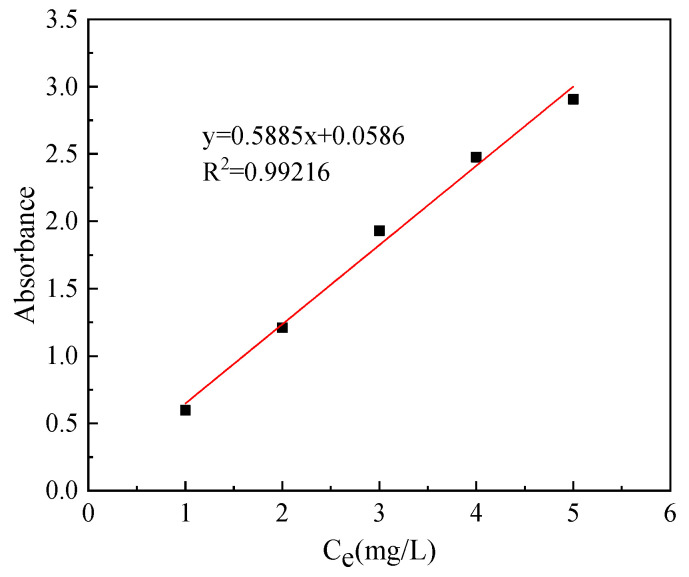
Standard curve of Naphthalene solution.

Naphthalene was dissolved in a certain volume concentration of ethanol solution to obtain the desired concentration of naphthalene solution. The absorbance of naphthalene solution with different concentrations was measured at 219 nm using a UV-2600 spectrophotometer, and the measured data were analyzed by linear regression [16]: Y_219_ = 0.5885x + 0.0586, and R^2^ = 0.99216, refer to Figure 10.

### 4.5. Adsorption Kinetics

To determine the type of naphthalene adsorption by biochar and the factors controlling the adsorption rate, we fit the adsorption data using the following three adsorption kinetic models. The kinetic constant k was used to determine the adsorption rate.

Pseudo-first-order kinetic equation [35]:

$$\frac{1}{q_{t}}=\frac{1}{q_{e}}+\frac{1}{q_{e}t}$$

Pseudo-second-order kinetic equation [36]:

$$\frac{t}{q_{t}}=\frac{1}{k_{2}q_{e}{}^{2}}+\frac{t}{q_{e}}$$

Webber and Morris equation [37]:

$$q_{t}=k_{i}t^{0.5}$$

where *q_e_* (mg/g) denotes the adsorption capacity of biochar at adsorption equilibrium, *q_t_* is the adsorption capacity at time *t* (mg/g), *k*_1_ (min^−1^), *k*_2_ [g/(mg · min)] and *k_i_* [g/(mg · min)] are the rate constants for pseudo-first-order kinetic, pseudo-second-order kinetic and Webber and Morris model.

### 4.6. Adsorption Isotherm

To investigate the relationship between the adsorption in solution and naphthalene at equilibrium, the following two adsorption isotherm equations were used to fit the adsorption data of biochar to naphthalene.

Langmuir adsorption isotherm equation [37]:

$$q_{e}=\frac{q_{m}bC_{e}}{1+bC_{e}}$$

Freundlich adsorption isotherm equation [38]:

$$q_{e}=K_{F}C_{e}{}^{\frac{1}{n}}$$

where *q_m_* is the monolayer saturation adsorption amount (mg/g), *b* is the Langmuir constant related to the heat of adsorption (L/mg), *K*_F_ is the Freundlich constant (mg/g) (mg/L)^1/n^, and *n* is the dimensionless heterogeneity factor.

## Figures and Tables

**Table 1:** Pore size structure of activated carbon.

Samples	S_BET_ (m^2^/g)	V_total_ (cm^3^/g)	V_mic_ (cm^3^/g)	V_mic_/V_t_ (%)	D_ap_ (nm)
WSC	50.25	0.0870	0	0	6.93
MWSC	1161.30	0.7128	0.3200	44.89	2.46
UWSC	579.94	0.3627	0.2384	65.73	2.50
EWSC	136.89	0.1049	0.0649	61.87	3.19

**Table 2:** Total Element Ratio of the Samples.

Samples	C/at.%	O/at.%	N/at.%	Na/at.%
WSC	89.28	9.24	1.43	0.05
MWSC	79.36	13.86	6.25	0.52
UWSC	86.87	8.84	3.48	0.8
EWSC	84.57	11.07	2.31	2.05

**Table 3:** C Content of the Samples.

Samples	Peak Position				Content %			
	C-C/C=C	C-O/C=N	C=OC-N	O=C-O	C-C/C=C	C-O/C=N	C=OC-N	O=C-O
WSC	284.81	286.28	287.58	289.48	71.86	13.19	5.81	9.14
MWSC	284.88	286.48	287.88	289.18	73.98	13.59	9.27	3.16
UWSC	284.92	286.28	287.38	289.18	69.94	9.43	8.89	11.74
EWSC	284.99	286.48	287.98	289.98	70.97	11.23	10.05	7.75

**Table 4:** O Content of the Samples.

Samples	Peak Position		Content%		nO=C/nO-C
	O=C	O-C	O=C	O-C	
WSC	532.16	533.68	91.32	8.68	10.53
MWSC	531.98	533.78	82.69	17.31	4.78
UWSC	531.99	533.64	72.05	27.95	2.58
EWSC	532.12	533.99	57.15	42.85	1.33

**Table 5:** N Content of the Samples.

Samples	Peak Position				Content %			
	Pyridinic-N	Pyrrolic-N	Graphitic-N	Oxidized-N	Pyridine-N	Pyrrole-N	Graphite-N	Oxidized-N
WSC	398.28	-	401.27	402.13	19.51	-	77.44	-
MWSC	398.58	400.36	401.51	402.74	29.01	59.74	6.23	5.03
UWSC	-	400.37	-	403.88	-	97.51	-	2.49
EWSC	398.57	400.47	-	-	64.04	35.96	-	-

**Table 6:** Naphthalene adsorption capacity of activated carbon.

t/min	q_e_ (mg/g)			
	WSC	MWSC	UWSC	EWSC
0	0	0	0	0
5	15.70	92.85	54.59	2.03
10	18.36	136.23	57.68	3.86
15	19.95	147.15	68.70	12.27
30	21.21	147.83	78.36	13.43
60	26.23	154.88	79.42	14.40
120	28.21	155.17	98.94	16.23
240	31.35	164.54	132.46	22.32
360	49.52	168.50	142.51	36.43
480	73.43	172.08	167.25	36.91
1440	90.63	192.85	214.98	48.21

**Table 7:** Kinetic Parameters of Pseudo-First-Order and Pseudo-Second-Order Adsorption Equations of activated carbon.

Samples	q_exp_ (mg/g)	Pseudo-First-Order			Pseudo-Second-Order		
		q_e_ (mg/g)	k_1_ (min^−1^)	R^2^	q_e_ (mg/g)	k_2_ (g/(mg·min))	R^2^
WSC	90.63	37.36	8.43	0.6674	95.88	0.00006	0.8932
MWSC	192.85	173.61	3.97	0.9176	192.68	0.00024	0.9976
UWSC	214.98	119.76	7.55	0.7142	219.78	0.00005	0.9776
EWSC	48.21	57.87	131.25	0.9509	52.06	0.00012	0.9666

**Table 8:** Parameters of Webber and Morris Equation for activated carbon.

Samples	k_3_ (g/(mg·min))	R^2^	k_4_ (g/(mg·min))	R^2^	k_5_ (g/(mg·min))	R^2^
WSC	1.6472	0.9004	0.6631	0.9990	1.7992	0.7922
MWSC	15.2565	0.6454	1.3014	0.8495	1.2873	0.9999
UWSC	7.7492	0.9533	6.8833	0.9973	3.5363	0.9612
EWSC	3.7988	0.8074	1.0437	0.9596	0.6490	0.9883

**Table 9:** Parameters of Adsorption Isotherm Model for Activated Carbons.

Samples	Langmuir			Freundlich		
	q_m_ (mg/g)	K_L_ (L/mg)	R^2^	K_F_ (L/mg)	n	R^2^
WSC	119.89	0.0296	0.9505	13.6139	2.4114	0.9781
MWSC	374.89	0.0120	0.9457	11.7329	1.5985	0.9607
UWSC	431.02	0.0111	0.9560	12.1649	1.5619	0.9674
EWSC	64.68	0.0238	0.9509	5.6380	2.1773	0.9964

## Data Availability

The data presented in this study are available on request from the corresponding author.
